# Three case studies of community behavioral health support from the US Department of Veterans Affairs after disasters

**DOI:** 10.1186/s12889-021-10650-x

**Published:** 2021-04-01

**Authors:** Tamar Wyte-Lake, Susan Schmitz, Reginald J. Kornegay, Felix Acevedo, Aram Dobalian

**Affiliations:** 1 Department of Veterans Affairs, Veterans Emergency Management Evaluation Center (VEMEC), 16111 Plummer St. MS-152, North Hills, CA 91343 USA; 2 Department of Family Medicine, Oregon Health & Science University, 3181 SW Sam Jackson Park Rd, Portland, OR 97239 USA; 3 Orlando VA Medical Center, 13800 Veterans Way, Orlando, FL 32728 USA; 4 VA Southern Nevada Healthcare System, 6900 N. Pecos Road, North Las Vegas, NV 89086 USA; 5 Division of Health Systems Management and Policy, University of Memphis School of Public Health, Memphis, TN USA

**Keywords:** Emergency preparedness, Behavioral health; disaster response, US Department of Veterans Affairs

## Abstract

**Background:**

Community disaster resilience is comprised of a multitude of factors, including the capacity of citizens to psychologically recover. There is growing recognition of the need for public health departments to prioritize a communitywide mental health response strategy to facilitate access to behavioral health services and reduce potential psychological impacts. Due to the US Department of Veterans Affairs’ (VA) extensive experience providing trauma-informed behavioral healthcare to its Veterans, and the fact that VA Medical Centers (VAMCs) are located throughout the United States, the VA is well situated to be a key partner in local communities’ response plans. In this study we examined the role the VA can play in a community’s behavioral health response using case studies from three disasters.

**Methods:**

This study investigated experiences of VA employees in critical emergency response positions (*N* = 17) in communities where disasters occurred between 2017 and 2019. All respondents were interviewed March–July 2019. Data were collected via semi-structured interviews exploring participants’ experiences and knowledge about VA activities provided to communities following the regional disasters. Data were analyzed using thematic and grounded theory coding methods.

**Results:**

Respondents underscored VA’s primary mission after a disaster was to maintain continuity of care to Veterans. The majority also described the VA supporting community recovery. Specifically, three recent events provided key examples of VA’s involvement in disaster behavioral health response. Each event showed VA’s integration into local response structures was facilitated by pre-existing emergency management and clinical relationships as well as prioritization from VA leadership to engage in humanitarian missions. The behavioral health interventions were provided by behavioral health teams integrated into disaster assistance centers and non-VA hospitals, VA mobile units deployed into the community, and VA telehealth services.

**Conclusions:**

Recent disasters have revealed that coordinated efforts between multidisciplinary agencies can strengthen communities’ capacity to respond to mental health needs, thereby fostering resilience. Building relationships with local VAMCs can help expedite how VA can be incorporated into emergency management strategies. In considering the strengths community partners can bring to bear, a coordinated disaster mental health response would benefit from involving VA as a partner during planning.

**Supplementary Information:**

The online version contains supplementary material available at 10.1186/s12889-021-10650-x.

## Background

### Disaster behavioral health

The current COVID-19 pandemic, while still underway, has already demonstrated the need for psychological interventions to manage the isolation, stress, and trauma stemming from the ongoing disaster [1–5]. While the scale of the event is unprecedented, interest in understanding the psychological consequences of disasters is not unique to the pandemic [6–10]. Generally, studies exploring the impacts of disasters on behavioral health have found increases in psychological distress in the short term, with the potential for some individuals to experience long-term psychiatric disorders such as posttraumatic stress disorder, depression, and anxiety [6–10].

To ameliorate the potential negative health effects to impacted populations, numerous early interventions have been explored [7, 11–14]. Some countries such as the Netherlands and Iceland use federally structured plans to implement disaster behavioral health interventions [12, 15]. And countries like New Zealand have invested heavily in developing extensive mental health programs in response to large scale disaster events [16, 17]. In the United States, federal entities strive to create resources and guidance on implementing behavioral health services after disaster, however, interventions are generally managed and delivered by state, territory, and local agencies [14, 18, 19]. Often, after federally declared disasters, the US Federal Government additionally provides funding through the Crisis Counseling Assistance and Training Program (CCP) to community behavioral health programs [8, 13, 20]. Yet the structure and content of interventions are left to the discretion of the implementing agency.

Regardless of the services provided, having a plan in place prior to a disaster can facilitate implementation [8, 11–13, 19, 21, 22]. Few publications detail the creation of a local disaster behavioral health response plans [15, 18, 20] or describe the process in which interventions were deployed following an incident [7, 10, 15, 20, 23, 24]. Common themes arising in studies exploring disaster behavioral health plans or interventions are the need for interdisciplinary teams and interagency collaboration [8, 12, 18–20] and strong community response [16].

### The United States Department of Veterans Affairs

One interagency partner often overlooked in the United States is the U.S. Department of Veterans Affairs (VA) Veterans Health Administration (VHA). Previous publications on VHA’s participation in collaborative disaster planning and preparedness efforts with local communities identified barriers to its involvement [25, 26]. One such barrier is that the community, and sometime even VHA employees, are unaware that VHA is tasked with planning for and acting to support “national, state, and local emergency management, public health, safety and homeland security efforts” [9, 27]. This responsibility to contribute to community efforts is called the VA’s Fourth Mission and is in addition to VHA’s duty to ensure continuity of services to veterans after a disaster.

The expertise of VHA’s 322,030 healthcare professionals and support staff who provide a range of services at its 1255 healthcare facilities spread throughout the U.S. and its territories makes VHA a valuable potential partner in disaster response [28]. In addition to inpatient and ambulatory medical care, VHA provides a variety of reintegration programs including trauma recovery and behavioral health services for Veterans and their families at its facilities and through community-based care at Vet Centers, Mobile Vet Centers, and college and university campuses [29]. How these services are applied in community response efforts has been detailed in the grey literature [28, 30–32].

This study highlights the potential role of local VA facilities in supporting local behavioral health activities after a disaster, and specifically, presents three exemplars of VHA integrating into communities’ disaster behavioral health response and providing behavioral health support to non-Veterans. The three VA facilities and the events they responded to are: (1) VA Pacific Island Healthcare System (VAPIHC), which is based in Honolulu, Hawaii but provides care to Veterans throughout numerous Pacific Islands. On October 24th, 2018, Super Typhoon Yutu made direct landfall on the Mariana Islands, a US Commonwealth with a nascent established VA tele-mental health clinic on the island of Tinian. This was the strongest typhoon ever recorded to strike the area, severely damaging or destroying many buildings and much of the critical infrastructure of Tinian [33]; (2) Orlando VA Healthcare System, which serves east central Florida, and encompasses 7 counties. On June 12, 2016, a domestic terrorist attack [34], targeted hate crime, and one of the deadliest mass shootings in the U.S. occurred at a local establishment, Pulse Nightclub. In a matter of hours, 49 people were killed and 53 were wounded before law enforcement breached the building and ended the violence [35]; and (3) VA Southern Nevada HCS (VASNHCS), located in and providing care throughout Las Vegas, Nevada. On October 1, 2017, the worst mass shooting in modern history took place at the Route 91 Harvest Music Festival on the downtown Las Vegas Strip. A gunman opened fire on a crowd of more than 22,000, killing 58 people and wounding 413 [36]. These cases provide examples of interagency partnerships and the implementation of collaborative responses for communities developing their own plans to address the behavioral health needs of their citizens during disasters, including the ongoing COVID-19 pandemic.

## Methods

### Study design

The results presented in this paper stem from a larger study broadly examining the role of local VA facilities in responding to regional large-scale disasters. This study used qualitative interview methods to elicit study participants’ experiences during disasters impacting the U.S. between 2016 and 2018 (see Table 1 for the full list of the disasters covered in the study, the impacted US states and territories, and VA entities affiliated with the impacted areas). Findings on disaster behavioral health functions were pulled as a subset of data and analyzed. The VA Greater Los Angeles Healthcare System Institutional Review Board (Los Angeles, California USA) approved this study.

**Table 1:** States, disasters, and VA facilities represented by the research

Disaster	Dates of Disaster	US States & territories Impacted	Affiliated VA entities impacted by the disaster
Pulse Nightclub Shooting	June 12, 2016	Florida	Orlando VA HCS VISN 8 VA OEM
Hurricane Harvey	Aug. 17 – Sept. 1, 2017	Texas; Louisiana	VA Texas Valley Coastal Bend HCS VISN 16 VA OEM
Hurricane Irma	Aug. 30 – Sept. 9, 2017	US Virgin Islands; Puerto Rico; Florida; Georgia; Alabama	VA Caribbean HCS VISN 8 VA OEM
Hurricane Maria	Sept. 16–30, 2017	US Virgin Islands; Puerto Rico	VA Caribbean HCS VISN 8 VA OEM
Route 91 Harvest Festival Shooting	Oct. 1, 2017	Nevada	VA Southern Nevada HCS VISN 21 VA OEM
California Wildfire Seasons	2017 & 2018	California	VA San Francisco HCS VA Northern California HCS VA Greater Los Angeles HCS VISN 21 VISN 22 VA OEM
Hurricane Michael	Oct. 7–11, 2018	Florida; Georgia; Virginia; North Carolina	Gulf Coast HCS VISN 8 VISN 16 VA OEM
Typhoon Yutu	Oct. 24, 2018	Northern Mariana Islands	VA Pacific Islands HCS VISN 21 VA OEM

*Note*: *VA HCS* Veterans Health Administration Health Care System, *VISN* Veterans Integrated Service Network. The national VA system is currently divided into 18 of these regional networks; VA OEM = Office of Emergency Management headquartered in Washington, DC

### Setting and sample

The full study sample was purposively chosen to represent individuals with emergency response roles critical to coordinating VA’s local response to disasters. Emergency management personnel at various levels of the VA were the first point of contact and, when applicable, identified additional individuals with critical response roles to interview. Additional respondents were recruited independently by the project team These facilities made up the broad recruitment sample. Due to some respondents covering multiple disasters or being deployed to disasters outside their normal service region, not all entities were included in the final sample.

### Data collection methods

Data were collected through semi-structured, 60-min telephone interviews between March–August 2019, using an interview guide developed for this study (see Additional file 1). Interviews were conducted individually with each respondent and led jointly by at least two of the authors. Interviews explored participants’ experiences and knowledge about VA activities in the community, specifically focusing on how VA networked and coordinated with non-VA community agencies. Interviews were audio-recorded, although one respondent declined to be recorded.

### Analysis plan

A total of 17 individuals were interviewed. However, five interviews did not indicate collaborating with non-VA entities and were therefore not included in the analysis. Due to their involvement in multiple disasters, two respondents were interviewed twice. At completion of the interviews, this resulted in 13 interview recordings being transcribed, and one set of interview notes (due to interviewee declining to be recorded), resulting in a total of 14 interviews that were analyzed with Atlas.ti (v.7) using a grounded theory approach. In phase one of analysis, one author reviewed all 14 interviews, using inductive coding to identify emergent themes in the data, and informed by extensive conversations about project findings held by the project team at the conclusion of each interview [37]. As a product of this process, a significant emergent theme [37] was the presence of interagency partnerships implementing collaborative responses to address the behavioral health needs of local citizens during disasters. A decision was made by the project team to narrow the focused coding analyses [37] to the way local VA facilities engaged in a behavioral response within their local community, in response to their respective large-scale disaster events.

In phase two of the analysis, the initial codes identified by SS were reviewed by TWL for consistency and agreement. Codes not deemed consistent to the focus topic were dropped. Additional grounded themes were confirmed by the team and applied to the data set [37]. Consensus on final codes was achieved, and one code list was finalized. In phase three of the analysis, the final code list was applied across all relevant interviews. The final code list had a focus on behavioral health response, including behavioral health activities, method of delivery, reactions of staff, and types of impacted community populations, but also included an identification of high level themes across all disasters, including VA expertise, integrating into local, established response activities, identification of local needs, and logistical challenges. Authors TWL and SS then independently coded each interview and resolved discrepancies by consensus.

## Results

All respondents played a substantial role in VA’s activities following the respective disasters. However, only 12 participants indicated the VAMC they supported collaborated with non-VA partners during the event in question. Included disasters ranged from widespread to geographically contained; weather-related to acts of violence; and direct impact on VA facilities ranged from none to significant. Though not all respondents described intensive engagement with the community following the event, all respondents described the importance of integrating into local, established response activities. This translated into involvement in community-wide drills and planning committees and following the lead of local incident command. Respondents indicated one of the areas where the VA could provide support to the community was in disaster behavioral health relief operations.

Activities described by respondents were often centered around tasks where the VA could reduce the caseload of other community agencies by identifying Veterans obtaining services in the community and meeting their needs regardless if they were previously enrolled in VA benefits. One key activity described by several respondents included outreach into local shelters. As one respondent explained, VA staff at shelters *“[distribute] fliers [that] outline that our counselors are experts in trauma, loss, and in readjustment. They also provide referrals to Veterans for a variety of services, including housing and employment. We also offered free counseling for all community members impacted*.”

Identifying where shelters were established and receiving authorization to deploy VA assets to those locations required coordination with local authorities. Multiple respondents mentioned connecting with emergency management running relief efforts to describe available VA resources and detail the services available to both Veterans and the community at large. In some of the events explored in this study, the non-VA authorities were unaware of what the VA could offer while others had pre-existing relationships that allowed for more transparent understanding of how the VA could support response efforts. One respondent went on to describe how the disaster that impacted their VAMC led to additional outreach to local jurisdictions and shelter coordinating agencies (e.g., the American Red Cross) to build relationships and understanding specifically of the behavioral health services the VA could deploy, if needed and approved.

Respondents noted there were specific benefits to conducting outreach in locations where other agencies provided services to the people impacted such as shelters and Local Assistance Centers. They noted that VA staff could more easily reach Veterans to enroll them, if eligible, into VA services and offer care to those who usually used non-VA health and mental health facilities, thereby supporting local agencies by reducing potential patient loads elsewhere. Additionally, by positioning resources at a central location, VA could more readily offer community members services as an extension of their work with Veterans. One specific resource identified as useful for Veteran and community support was Mobile Vet Centers, which have the primary goal of providing social work and mental health services to Veterans. In cases where respondents mentioned this resource, they underscored that non-Veteran community members who requested services in the first days after the disaster were never turned away.

### Three community profiles

Three disaster events described by respondents distinctly highlighted cases where the VA was deeply involved in the local community’s disaster behavioral health response. Each event showed VA’s integration into local response structures was facilitated by pre-existing emergency management and clinical relationships, as well as prioritization from VA leadership to engage in humanitarian missions to support the community.

#### Telehealth in Tinian, Mariana Islands

Prior to Super Typhoon Yutu impacting the Mariana Islands, the VA Pacific Island Healthcare System (VAPIHC) established tele-mental health services on the island of Tinian. These services were located at a non-VA owned healthcare clinic using pre-positioned VA telemedicine equipment and coordinated with the clinic director and staff. Typhoon Yutu devastated the island and led to many Tinian healthcare clinic employees losing their homes. The clinic with VA tele-mental health equipment became a temporary housing site for staff as it was undamaged by the storm. The clinic director realized that in addition to sheltering needs, employees also experienced significant trauma. However, there were limited mental health resources on the island. Once VA became aware of the need, it worked with other federal agencies to manage the logistics of implementing services that took advantage of pre-positioned VA resources.

“… it was a relationship that we had with [the US Department of Health and Human Services] (HHS) and a relationship that we had with the folks on Guam and Saipan … we have a lot of relationships going on. So, we knew that we had that telehealth equipment. We also knew that Tinian was … hit pretty hard. And that there was a lot of grief. And so I can’t say how it totally emerged, but there’s so many relationships and there’s so much communication during an emergency.”

Respondents reported it was initially challenging to identify whether VA could provide mental health services in the community and how the services would be funded. Staff at all levels of the VA worked with the Federal Emergency Management Agency (FEMA) and HHS to get official authorization as well as receive federal funding for VAPIHC to provide time limited tele-mental health interventions to clinic staff on Tinian. VAPIHC Tele-mental Health Hub coordinated with the local clinic director to inform employees about available services and utilized technology onsite to provide weekly support groups for 13 health center employees.

#### Director’s 50 in Orlando, Florida and the pulse nightclub shooting

The Orlando VA Healthcare System (OVAHCS) houses a unique emergency response team “The Director’s 50.” Made up of multi-disciplinary VA healthcare workers, including mental health professionals (i.e. psychologists, psychiatrists, mental health nurses), the Director’s 50 can deploy a team of up to 50 volunteers within 2 hours to areas throughout the region when authorized by the Orlando VAMC Director. As described by one respondent, the mission of the team is,

“to provide an immediate gap fill to an emergency before VA can get its assets organized and into a formal support and response role. So the team is multi-disciplinary and multi-functional with its capabilities, so that it can immediately address the needs of the emergency response until VA can formalize how it’s going to provide their support to the community.”

The Director’s 50 includes interdisciplinary clinical and service support training for all members such as triage and treatment services, mental health intervention, peer counseling, and psychological support to trauma. Through participation in community-wide exercises and drills, the Director’s 50 has built versatile capabilities and strong relationships with local emergency management agencies and area hospitals.

In response to the Pulse Nightclub Shooting, VA Central Office requested OVAHCS to deploy the Director’s 50 to provide VA resources and support the community’s response. The team activated their mass notification system to alert their nearly 100 volunteer members and quickly assembled an initial response team of about 15 clinical, mental health, and support professionals within 1 hour. Respondents noted having internal approval can speed up the process of deploying teams. In general, to distribute VA resources into the community, a federal disaster declaration is required to initiate the Robert T. Stafford Disaster Relief and Emergency Assistance Act or where the HHS Secretary has activated the National Disaster Medical System, both of which grant VA the ability to provide assistance. Therefore, respondents noted a need to balance expectations of leadership to help quickly, while also ensuring VA resources were legally allowed to be used in the response.

One thing that facilitated OVAHCS’s integration into the local response system was a pre-existing relationship with the City of Orlando’s Office of Emergency Management and the Central Florida Medical Disaster Coalition, which facilitated the Director’s 50 integration into the city’s response and allowed them to report to the victim reunification center. The team was tasked.

“to be the initial communication to the family members for those victims that actually passed away. So, 49 victims, our team was assigned to go ahead and be the initial contact to let them know that their loved ones had passed, and to begin the coordination for services, grief counseling and victim advocacy, you know, to help them prepare the initial points of piecing together their lives after being notified of such tragic events.”

Accordingly, the initial multi-disciplinary team narrowed its focus to mainly members with mental health expertise. Over the next 2 weeks, the team worked with the community, helping to manage vigils and gatherings for the public, and continuing grief counseling and mental health support for the whole community, including providing peer behavioral health support to municipal first responders. Since this act of violence targeted people who were Lesbian Gay Bisexual Transgender Queer (LGBTQ) frequenting Pulse Nightclub, not only were relatives of victims or survivors from inside the building affected, but the entire LGBTQ community felt the traumatic impact of the shooting. One respondent described the importance of providing mental health support from multiple community agencies when a disaster of this magnitude occurs,

“And they [the people who were at the shooting] truly needed a place, and this is why we were there for greater than just the 24-48 hours of initially identifying the people who was killed during the shooting, you had everyone that was inside of the club who were seeking a place where they could go and receive the care and support that they needed as well. And obviously, you know, this is something that is an endemic issue with healthcare as a whole, is the access to mental health counseling and services. So VA, as well as some other partnering mental health organizations were able to supply that need right there at the site where they were doing victim notification or victim reunification and family support. We were able to do that.”

One respondent noted a key point to remember about the Director’s 50, “they are all volunteers...And these people will go—you know, 24 hours a day, day in and day out, to execute that mission. And we have to think about team resiliency.” This included caring for team member’s well-being by rotating staff and providing and attending to the mental heath of one another. As described by one respondent,

“Because when it was all said and done, the team was very affected by what they had to do. You know, just imagine hearing—you know, overwhelming grief for every one of the 49 victims’ families that would show up. And the team took that burden on … and I will tell you, to this day, it still affects the people who went and supported that mission. And they really—those who supported that mission have a greater reverence for what we do now, as a team. So you’d never have to ask them to—whether they are going to support anything related to the Director’s 50. That comradery that’s there, they won’t let their own kind of—go into the bowels of despair like that, alone.”

#### Integrating into community response in Las Vegas, Nevada after the route 91 harvest festival shooting

As a large city with many national and international visitors, respondents described Las Vegas as having a very centralized emergency response structure. Relationships between VA Southern Nevada HCS (VASNHCS) and local response agencies and area hospitals were described as “tightknit” with great working relationships where organizations plan and prepare for disasters together. As one respondent put it,

“what I do know is my community. I know my community partners. I know what they have, what they don’t have, they know what I have, what I don’t have. And that’s what makes us so resilient. That’s community.”

Although located too far away from the Las Vegas Strip to actively receive injured victims when the shooting occurred at the Route 91 Harvest Music Festival, VASNHCS activated its Hospital Incident Command System so it could actively participate in the community’s response and organize efforts. A Multi-Agency Coordination Center (MACC) organized the response activities, and respondents underscored the value of both pre-existing relationships and an understanding of the county’s emergency response structure. As explained by a respondent,

“You can’t wait for your community to ask you. You have to be on the forefront and know what they need. And you only do that by knowing your community. You know, I spent probably as much time in my community as I do in my medical center. A lot of the time, it’s my own time, but again, it builds that relationship that when they’re updating their mass casualty plan, one of the people they’re calling is [me].”

This previous collaboration, as well as being present at the MACC, allowed VASNHCS to identify community needs that it could address.

As news of the shooting spread, VA leadership tasked VASNHCS with deploying staff into the community. However, it was challenging to balance the push from VA to deploy with continuing to respect established local coordination structures. VASNHCS maintained a presence within the Medical Area Surge Command of the MACC to offer resources and expertise, waiting for requests, instead of directly deploying assets outside of the established system.

In the immediate response, VASNHCS assisted with managing fatalities. It offered morgue space to the county and initiated the mass fatality plan to increase morgue capacity. This provided the county and partner hospitals space for victims until they could be processed, and families could claim them. Additionally, VASNHC offered a Psychological First Aid (PFA) team.

Initially, VASNHCS deployed their PFA team to the community’s family reunification center. The team was composed of social workers, psychologists, psychiatrists, administrators (as support staff), canteen services (for water and snacks to sustain clients and staff), and the medical center’s Chief of Staff. As the situation evolved, the MACC received requests from local agencies for psychological assistance and VASNHCS transitioned to directly integrating into area hospitals.

Three Las Vegas hospitals received the bulk of the injured or dead and recognized the need for psychological interventions with their staff. Due to their close relationships with other hospitals, one respondent explained that they were familiar with the Employee Assistance Program (EAP) at these hospitals. The respondent knew it would take time for the EAP to arrive onsite and they would most likely focus on clinical staff involved in directly treating the injured. Therefore, VASNHCS developed a three-pronged approach to complement EAP services at the receiving hospitals. Firstly, the PFA team provided what one respondent called “*trauma therapy*” to hospital staff, regardless whether they worked the night of the shooting. The assistance extended beyond clinical staff to non-clinical departments, such as environmental services/housekeeping, whose staff were also impacted through their response roles.

Respondents reported one of the reasons their response in the hospitals was so successful was that the team was multidisciplinary, allowing staff from different departments to talk to people in similar positions, which was valued by the recipients.

“So for example, we have a nurse that’s trained in trauma, psychological first aid. So they want the nurses at [the hospital with a patient surge], they want to talk to our team. They were still processing. But when we brought our nurse into the ward, they were more than willing to open up to her, because she was one of them. She was part of their tribe. So we try to match our tribe to their tribe, and that’s why we were successful.”

Secondly, the VASNHCS team worked with victims of the shooting, providing PFA and social work services. Thirdly, they integrated with family members of patients at the hospitals and provided them items that they did not otherwise have because they were visitors to Las Vegas. Examples included coordinating free transportation to and from hospitals and hotels, connecting them to local mortuary services, and providing information about how to access services when they returned home.

The PFA team ran for 24 h a day, for 7 days in those three impacted hospitals. To balance VA patient care with the community response mission, VASNHCS staff volunteered shifts outside of their normal work hours. One respondent described the overwhelming desire of VA staff to help their community.

“And while it didn’t impact our staff or our clinics, or our patients, it impacted our community. I think another thing that still amazes me to this day, was the outpour of our staff and what I mean by that is they were coming out of the woodwork to support. We had more volunteers working an eight-hour shift and then coming in [to volunteer] at five o’clock or four o’clock and working to midnight to two in the morning and not go home until four or five in the morning, and then go to work the next day, because we didn’t want to impact our patient care. And they were doing this out of their—you know, because they care. They care about the community, they care about the event, they care about the people. And then at the end of the day, you know, we had more volunteers than we had placements, because we did not want to overwhelm the health systems with all of these VA personnel.”

However, with new volunteers each shift, a key lesson learned was to have a daily team debrief. As people changed daily, a debrief provided key information and a running tally of support being provided to save time and avoid reinventing the wheel identifying contacts or systems already developed.

Another lesson was that preparedness requires ongoing maintenance. The importance of ongoing preparedness was underscored when VASNHCS realized that leading up to the shooting, they had reduced their focus on PFA training. As described by one respondent, “*We noticed that we need that continuous* [psychological first aid] *training, that we need continuous exercising, and it’s not an easy fit, to send a bunch of people to someone else’s hospital or an area to do that kind of service*.” They also realized the first wave of personnel went into community hospitals without basic supplies they needed to provide services, including basic items such as pens, PFA guides, and informational brochures.

Three months following the shooting, the VASNHCS Emergency Manager, working with the Chief of Social Work hosted a lunch for staff who volunteered to thank them for their involvement. During that event, they realized volunteers were not only impacted by the event itself, but also by their time providing support in the community. They therefore created a forum to again gather staff who had deployed at the 6 month and 9 month marks to eat and talk about the impact of the event on the healthcare system and themselves. On the 1 year anniversary, management had a special event for the volunteers,

“we actually had people from the community that we supported coming in and they broke bread with our team and what they did was, they talked about what the impact of the VA Southern Nevada Healthcare System was going into that event, and how we helped them bridge the gap [of mental health support] that was crucial at that time, and how appreciative they were to our cause and our Clark County Office of Emergency Management gave all our staff that responded T-shirts that said Vegas Strong, because they wanted them to know that we—they appreciated the work that we did for them to support our community.”

## Discussion

The need for attention to the psychological well-being of individuals during and after a disaster has been well proven as disasters have been found to be associated with both short and long-term symptoms and disorders [6–10, 38]. Although in some parts of the world there are federally structured plans to implement disaster behavioral health interventions, in the United States there is a patchwork system that often relies on support and resource allocation from a multitude of agencies [8, 13, 14, 18–20]. In this study we examined the role the VA can play in a community’s disaster relief effort and highlighted the opportunity for VA to support behavioral health response focusing specifically on case studies from three disasters.

Essential to effective emergency management is an understanding of, and engagement with, available resources in a local community. This is of particular importance when considering complex individual and group needs such as behavioral health support. VA Medical Centers can be seen as challenging partners to work with because they are both a federal entity and a local healthcare facility [26]. However, in the case of Super Typhoon Yutu, the federal positioning of the VA and its connection with HHS and FEMA facilitated the deployment of VAPIHC virtual resources. The regional respondents who supported the VA disaster mission in Tinian described how preexisting relationships with federal partners facilitated authorization and funding.

Another potential challenge to incorporating the VA into response efforts is that prior to offering services, VA leadership must balance the mission of the agency with community needs, without contradicting the restrictions of the Stafford Act. In all three presented cases, VA’s behavioral health support was not formally included in a city or county response plan, and yet pre-existing relationships between key stakeholders facilitated the provision of VA behavioral health services to support identified community needs. Respondents also described participating in interagency coordinating groups, response trainings, and exercises before the disaster. These activities aided in a deeper understanding of the response structures each partner operated under and encouraged strong rapport between agencies.

Relationships between VA emergency management and local emergency management proved invaluable as VA staff understood that services should not be provided without first engaging local response coordinators. All VA facility leadership and emergency managers are required to be trained in the Incident Command System (ICS) and National Incident Management System (NIMS), which are the coordinating structures all U.S. response agencies work within [39]. As a health care provider at a national level, VHA falls within the operations section Essential Support Function (ESF) 8: Public Health and Medical Services to support the Department of Health and Human Services [40]. Local jurisdictions may also connect with VAMCs through ESF 8 representation. For example, the VA has provided significant support to communities impacted by COVID-19. As of July 8, 2020, VA provided more than 330,000 pieces of Personal Protective Equipment (PPE) in support of the Fourth Mission, as well as hand sanitizer, laundry support, test kits and testing support, and webcams for use with existing equipment to state and local facilities. In addition, VA has admitted 279 non-Veterans to VA Medical Centers because of the pandemic [27]. Much of this coordination was done through ESF 8 coordination at a local or national level.

While respondents did not go into detail about their participation in the emergency management structure, some participants described their VA’s roles within emergency operations as liaisons. Groups such as this could report to either the planning, operations, or command sections within the ICS. Group supervisors would most commonly report to the operations section chief, likely through branch directors, given that the focus of the work would be more on specialized functions as needed for tactical operations. Regardless of where they fit, their presence at emergency operations centers and command posts facilitated communication to allow for VA’s integration into incident action plans. Particularly during the response to mass casualty events in Orlando and Las Vegas, understanding the local response network and then proffering available services was essential to avoid confusion or duplication of activities. By working within the established coordination centers, VA’s efforts were effectively integrated into the greater community behavioral health response and were deployed to points of greatest need. Although a detailed understanding of the integration of VA activities into ICS structures fell outside of the scope of this work, future assessment of the integration of VA representatives into local, state, and/or regional ICS structures could help clarify roles and identify which section liaisons best support (e.g., operations, planning, logistics) [40].

VA is increasingly strengthening partnerships with agencies that provide behavioral health services to Veterans and their families who use non-VA community-based care [41]. In each case example, the primary support provided by VA to the community was the provision of behavioral health services in response to an identified need. Respondents described this as being due, in large part, to the recognition of VA’s expertise in trauma and post-trauma treatment, thereby allowing these resources to come to the forefront. While not mentioned by the respondents, an additional value that VA providers add to disaster behavioral health responses is their exposure to and understanding of the unique needs of various populations throughout their communities. In addition to ensuring care is culturally competent to the unique identity of being a Veteran, VA staff must respect the diversity of Veterans themselves. Just like the U.S. population at large, Veterans represent a range of ages, races, genders, sexual orientations, socioeconomic statuses, etc. and mental health services must be considerate of this diversity. The VA recognizes this and offers training to providers to understand and respect their patients’ unique needs [42]. Working with a variety of populations preposition VA staff to have a deeper understanding of the post-disaster needs of the wider community.

Two of the case examples described in this study especially bring to the forefront the importance of disaster behavioral health response planning and implementation teams understanding unique experiences of community members. Super Typhoon Yutu directly impacted an archipelago housing a majority Asian and/or Pacific Island population. The Pulse Nightclub shooting, while a terrorist event, was a targeted hate crime intended to inflict violence on the LGBTQ community. Disaster behavioral health interventions for these affected groups not only need to take into consideration the importance of cultural competency but also the potential of re-traumatization and distinct population mental health needs.

In the Northern Mariana Islands, while there is a mix of ethnic groups (Filipino, Chamorro, Chinese, Carolinian, Korean, Palauan, etc.), many either identify as or are categorized more broadly as Asian and/or Pacific Islanders. Although there are more than 1.4 million people who are considered Pacific Islanders living the in the U.S., there is a dearth of information on the mental health of this population [43]. Similarly, the prevalence and incidence rates of mental illness in the Mariana Islands is not well studied [44]. Some sources attribute this lack of understanding to a disproportionate underuse of mental health services [43]. However, Asian and Pacific Islanders within the U.S. and those territories affiliated with it often experience transgenerational trauma, discrimination, continued loss from colonization, historical trauma, and mental health stigma which can impact psychological wellbeing and help seeking behavior. Additionally, cultural elements (collectivism, reverence for the past, hierarchical social order, etc.) of this population are important to understand when providing behavioral health services [43, 45]. One of the reasons respondents indicated that the VA was asked to provide assistance following Typhoon Yutu was the lack of availability of mental health services in Tinian. The established VA telehealth technology increased accessibility to behavioral practitioners from VAPIHC who most likely were experienced working with Asian and Pacific Island populations since more than 55,000 Veterans who identify as this ethnicity live in Island Areas or Hawaii [46, 47].

The Pulse Nightclub Shooting was a terrorist driven hate crime targeting individuals who identified as LGBTQ. Members of this group often experience discrimination, stigma, and trauma throughout their lives. Discrimination and heterocentric health and mental health practices can marginalize this population and impact help seeking behavior [48, 49]. This is of particular concern as individuals who are LGBTQ face numerous mental health disparities with a higher likelihood of experiencing depression, anxiety, substance misuse, and suicide attempts. The shooting not only targeted LGBTQ people but it also took place during Latin Pride Night meaning many of the victims and casualties were LGBTQ Latinx. The resulting psychological impacts of the Pulse Nightclub shooting on those directly impacted, people who are LGBTQ Latinx, and individuals in the wider LGBTQ community have been investigated and show experiences of trauma and impacts on perceived safety [50]. At the time of the shooting, the Orlando VAMC had established relationships with LGBTQ local mental health services and had staff knowledgeable in the needs of this community [51]. In fact, in the recent past, the VA has increased its efforts to ensure Veterans who are LGBTQ receive the highest quality patient-centered care possible [52]. Mental health services in particular have bolstered recognition of the complex needs of these Veterans [52].

All three cases demonstrate innovative ways VA can provide behavioral health support outside of their facilities, i.e., via telehealth capabilities across an ocean and into a healthcare clinic, teams of mobile units reaching directly into the community to support victims, victims’ families, and the community at large, and finally by incorporating PFA teams directly into hospitals to support staff, patients, and patients’ families. This flexibility across sites to address different needs and populations while using varying available infrastructure support, is paramount to any local jurisdiction’s ability to meet on the ground needs following a disaster. It demonstrates the variability between VAMCs and the importance of local disaster behavioral health planning teams to pre-identify resources to assess local capacity. Plans can then be developed that access and deploy the tools/skills of interdisciplinary and interagency teams. Building processes to deploy local health and mental health practitioners can lead to more rapid implementation of interventions and help ensure the diversity of the impacted community is recognized and respected. Additional studies focused on how communities develop disaster behavioral health plans could provide insight into which agencies are involved and how they collaborate. It may also be useful to assess whether and how these plans are implemented to identify best practices.

In addition to the people directly impacted by disasters, respondents underscored the importance of offering support to responders as well. There is growing recognition that health care workers are themselves front-line response workers who may be psychologically impacted when caring for others, leading to a growing emphasis on the importance of selfcare and employee wellbeing [39–42]. In all three case studies, behavioral health support was, at least in part, directed toward healthcare workers. In the case of Las Vegas, a respondent highlighted the advantage of having behavioral health support come from individuals who understood the culture of the population they were helping, e.g., nurses supporting nurses. Further, respondents in Las Vegas and Orlando highlighted the importance of supporting deployed behavioral health team members. They detailed actions to maintain staff well-being by having rotating shifts, encouraging peer support, and facilitating gatherings for staff to publicly thank them for their efforts and allow them to address their experiences together as a group. Understanding the needs of healthcare and behavioral health personnel and building support networks into response frameworks can help better sustain and strengthen the overall response process.

A primary limitation of this study is that interviews were conducted up to one and a half years after the disasters described, potentially impacting recall. However, multiple interviewees corroborated the information presented for each of the case studies. Another limitation is that this study focused exclusively on the experiences of VA employees fulfilling mission requirements and their description of instances where VA acted in support of the Fourth Mission. Very few of the respondents directly provided the behavioral health interventions. These perspectives could provide deeper understanding of the interventions themselves as well as the impacts they may have on practitioners. Neither community members nor coalition partners were interviewed in this study. Future research would benefit from both interviewing non-VA participants to explore additional perspectives and gain greater insight on how local jurisdictions experienced collaborating with VA representatives and exploring alternative approaches to mental health units within and outside VA to examine whether and when different approaches may be preferable.

## Conclusion

As the largest integrated healthcare system in the United States, VA can play an important role in disaster response across the country. As recognition of VA’s expertise in behavioral health grows, particularly around trauma and post-trauma treatment, VA should be considered a strong potential partner in behavioral health responses. Local VAMC staff are part of the community in which they live and the Veterans they serve are a microcosm of the larger population of the U.S. As the respondents in this study showed, there is a deep desire by VA staff to provide support following a disaster if they are able. Anticipating potential behavioral health concerns, and having a plan to address them, can foster community disaster resilience. While these plans may be different for each jurisdiction, they can be strengthened by identifying and incorporating a range of partners. Having preexisting relationships where VA’s capabilities are known before a disaster occurs can facilitate the rapid deployment of VA resources into identified areas of community need. The case studies presented demonstrate the flexible nature of these resources. By extending knowledge about innovative ways to share behavioral health and other resources in a disaster response, communities and healthcare coalitions can be better prepared to engage collectively and rapidly mobilize essential assets to support the wellbeing of those who need it most.

## Supplementary Information

**Additional file 1.** 2017 Disaster Qualitative Study: Collaboration Project Interview Guide. Interview guide utilized during project’s semi-structured interviews.

## Data Availability

The datasets used and/or analyzed during the current study are available from the corresponding author on reasonable request.

## Abbreviations

CCP: Crisis Counseling Assistance and Training Program
VA: U.S. Department of Veterans Affairs
VHA: Veterans Health Administration
VAMC: VA Medical Center
VAPIHC: VA Pacific Island Healthcare System
VASNHCS: VA Southern Nevada HCS
VA HCS: Veterans Health Administration Health Care System
VISN: Veterans Integrated Service Network
VA OEM: Office of Emergency Management
HHS: US Department of Health and Human Services
FEMA: Federal Emergency Management Agency
LGBTQ: Lesbian Gay Bisexual Transgender Queer
MACC: Multi-Agency Coordination Center
PFA: Psychological First Aid
EAP: Employee Assistance Program
PPE: Personal Protective Equipment
