# Supplementary material for: Three case studies of community behavioral health support from the US Department of Veterans Affairs after disasters
Source: BMC Public Health. 2021 Apr 1;21:639. doi: 10.1186/s12889-021-10650-x (PMC8015747; doi:10.1186/s12889-021-10650-x)
Supplement: Supplementary file 1 — Additional file 1. 2017 Disaster Qualitative Study: Collaboration Project Interview Guide. Interview guide utilized during project’s semi-structured interviews. [file 12889_2021_10650_MOESM1_ESM.docx]

General Information

*Here are a few background questions.*

1. You were selected for this interview based on your experience during the [disaster event]. We will be going into more detail during the interview, but for now, can you start by briefly describing:
   1. Your position at the VA
   2. Were there other disaster events over the course of the last year where your incident command system was activated?
   3. Your role in the [disaster event]
      1. Were you part of the incident command staff? What was your position (incident commander, operations section chief, liaison officer, etc.)?
      2. Who in your VAMC was directly responsible for fielding external resource requests?
   4. Could you tell us briefly how your healthcare system was impacted?

Providing support to the Community

*We know there was a lot of different activities happening in response to XX event. We are trying to better understand if and how local VAMCs work with their community during times of disaster when their facilities are also impacted. We would like to hear more about how your VAMC was involved, if at all, in supporting any identified humanitarian needs in the local community (e.g. providing water, food, transportation).*

1. Did your VA provide resources, services, and/or support to help meet any humanitarian needs in the community?
   1. In what ways?
      1. Prompts: water, N-95 masks, amenity kits, etc.
   2. With which non-VA entities did you work as part of your response in the community? (Seek specific examples)
      1. Federal agencies
      2. Non-federal agencies
         1. Red Cross
2. What prompted your activities within the community?
   1. Were services/support requested by another agency? By whom?
   2. Did you or your facility offer support/services without being requested?
   3. How did you identify any unmet needs in the community?
3. Were there informal or formal mechanisms for resource requests in place prior to the disaster?
   1. Examples: MOUs, coalition requests, WebEOC requests
4. Were there external agencies that were particularly helpful in facilitating your activities in the community? (Examples: coalitions, FEMA – which support collaboration)
5. What obstacles did you face when trying to collaborate with other agencies?

Receiving support from the community

1. Did your VAMC need to request any resources from non-VA agencies? By non-VA we mean local, state and other federal agencies as well as private sector organizations.
   1. What were they? From whom? How?

CBOC vs. VAMC activities within the community

*We would like to speak a little bit about any potential differences between your ancillary VA facilities (e.g., CBOCS, ACCs, Vet Centers, etc.) and the VAMC’s disaster responses.*

1. Are there differences in the types of services shared (or requested to be shared) with the community in the areas where your CBOCs are located? Are there ways in which the relationships with the surrounding community are different between CBOCs and VAMCs?
   1. Did CBOCs provide more humanitarian services to the community than the VAMC?
   2. Did CBOCs need more support from the community post-disaster?

Balancing VA Priorities

1. Did the VA get resource requests from the community that you could not fill?
   1. Was there a need to prioritize VAMC/CBOC resources?
      1. What considerations went into the decision-making process?
      2. When were those decisions made?
      3. Who was involved in making the decision on how to prioritize care to Veteran patients, staff, staff or patient families, and other community members?

Future Preparedness and Response Efforts

1. How could/did interactions with the community change your relationships for future situations?
2. Is there anything you wish you could have done beforehand? Or done more of?
3. Will there be any changes to the allocation of supplies/resources specifically to CBOC preparedness/response?
4. Are there any changes you’ve made post-disaster to policies/procedures/agreements?

Final Questions

1. Are there any additional lessons learned you would like to share? They do not need to be specifically related to collaboration.
2. Are there specific things you would like to share with other emergency managers that you wish you had known before this event?
3. Would you be able to send your healthcare system’s After Action Report to us? We would like to make sure we have a comprehensive understanding of your response.
